# Telomere Instability in Lynch Syndrome Families Leads to Some Shorter Telomeres in *MSH2+/-* Carriers

**DOI:** 10.3390/life10110265

**Published:** 2020-10-31

**Authors:** M. Carmen Garrido-Navas, Frances Tippins, Julian Barwell, Jonathan Hoffman, Veryan Codd, Nicola J. Royle

**Affiliations:** 1 Department of Genetics and Genome Biology, University of Leicester, Leicester LE1 7RH, UK; frances.tippins@nhs.net (F.T.); Julian.Barwell@uhl-tr.nhs.uk (J.B.); 2 Liquid Biopsies & Cancer Interception (LiqBiopCI) Group, Junta de Andalucía de Genómica Investigación Oncológica, GENYO–Centro Pfizer–Universidad de Granada, 18016 Granada, Spain; 3 Universidad Internacional de la Rioja, 137, 26006 Logroño, La Rioja, Spain; 4 Clinical Genetics Unit, Birmingham Women’s Hospital, Birmingham B15 2TG, UK; jonathanhoffman@nhs.net; 5 Department of Cardiovascular Sciences, University of Leicester, BHF Cardiovascular Research Centre, Glenfield Hospital, Leicester LE3 9QP, UK; vc15@leicester.ac.uk

**Keywords:** telomeres, DNA mismatch repair, *MSH2*, *MLH1*, Lynch syndrome

## Abstract

Lynch syndrome (LS) is an inherited predisposition to early onset of various cancers, caused by mutation in a DNA mismatch repair (MMR) gene. In heterozygous MMR^+/−^ carriers, somatic mutation, loss or silencing of the wild type allele increases the mutation rate, facilitating the initiation of MMR-defective cancers. These cancers are characterized by instability at short tandem repeats (STRs) and in telomeric DNA. We have investigated telomere length in saliva DNA from LS and control families, using single telomere analysis at XpYp and 12q and by qPCR to measure total telomeric DNA. Single telomere analysis showed a trend for shorter XpYp telomeres in *MSH2^+/−^* carriers compared to *MLH1^+/^*^−^ carriers or controls, but this was masked in the comparative analysis of total telomeric DNA. Comparison of age-adjusted telomere length within families showed that neither *MSH2^+/−^* or *MLH1^+/−^* children had consistently shorter or longer telomeres than their MMR^+/−^ parent, indicating the absence of an inter-generational effect on telomere length. Unexpectedly however, wildtype children in families with *MSH2* mutations, had significantly longer XpYp telomeres than their MMR^+/−^ parent. Altogether our data suggest that MMR insufficiency, particularly in *MSH2^+/−^* carriers, increases telomere instability and somatic cell turnover during the lifetime of LS mutation carriers but has minimal consequences for telomere length in the germline.

## 1. Introduction

Telomeres are the essential capping structures of chromosomes with well-established roles in ageing and cancer. Short telomeres activate a DNA damage response and cells with one or a few dysfunctional telomeres enter a senescent state. Cancer cells usually acquire mutations that allow them to bypass this replication barrier but must eventually activate a telomere maintenance mechanism [1,2,3]. Inheritance of a mutant allele at a DNA mismatch repair (MMR) gene causes Lynch syndrome (LS), a predisposition to early-onset of a variety of cancers with microsatellite instability [4]. Heterozygous carriers of *MLH1* or *MSH2* mutations show a higher level of instability at short tandem repeat (STR) loci (also known as microsatellite instability, MSI), with a bias towards deletions, than seen in their non-carrier relatives. This shows that heterozygous MMR^+/−^ cells have a limited capacity for MMR arising from haplo-insufficiency [5,6,7].

Telomeric DNA comprises tandem arrays of (TTAGGG)_n_ often interspersed with sequence-variant repeats at the proximal end of the telomere [8]. Thus telomeric DNA shares some features with STRs that, as stated, are unstable in MMR deficient cells primarily due to slippage on the repetitive DNA during replication [9]. There are additional obstacles that cause the replication fork slow or stall on telomeric DNA during S-phase. These include G-quadruplex structures that form on the (TTAGGG)_n_ strand and the requirement to unwind the t-loop capping structure. TRF1, an essential component of shelterin, is required for the efficient replication of telomeric DNA as it facilitates G-quadruplexes removal and prevents the manifestation of fragile telomeres [10]. We have shown that telomeric DNA is unstable in LS colorectal cancers (CRCs) and tumor derived cell lines that lack a functional *MSH2* gene, resulting in frequent gains and losses of telomeric repeats [11,12]. Telomeres also shortened faster in a primary human cell line with depleted MSH2 protein [13]. It is thought that the instability of telomeric DNA in MMR defective cells is associated with replication slippage, but whether this is also influenced by G-quadruplex formation and replication fork stalling is currently unknown.

It has been proposed that LS families show genetic anticipation, with the first cancer appearing at an earlier age in successive generations. This hypothesis is controversial [14] with some studies showing evidence that supports genetic anticipation, in particular in families with *MSH2* mutations, while data from other studies indicating that such observations may be explained by other factors [15,16]. The controversy persists partly because there is inadequate evidence for a biological mechanism that could cause genetic anticipation in LS [14]. Genetic anticipation, associated with inter-generational telomere shortening, is seen in various forms of dyskeratosis congenita (DC), for example in families with autosomal dominant DC caused by mutations in the telomerase RNA component (TERC) [17], and other short telomere disorders [18]. However, it is not known whether a subtle effect on telomeric DNA, associated with MMR deficiency, could contribute to genetic anticipation in LS [19]. In this study we investigated whether *MSH2*^+/−^ and *MLH1*^+/−^ carrier status affects telomere length within LS families. We did not find evidence of an inter-generational effect on telomere length between MMR^+/−^ parents and children. In contrast, single telomere length analysis (STELA) showed that wild type (WT) children in LS families tended to have some longer telomeres than their MMR^+/−^ parent and siblings, particularly when *MSH2* was mutated. Overall the data support the hypothesis that MMR insufficiency, particularly when *MSH2* is mutated, increases telomere instability and subsequent cell turnover in somatic tissues but has little consequence for telomere length in the germline where telomerase is active.

## 2. Materials and Methods

Saliva samples were collected from adult individuals (18 years or older) in control and LS families. All subjects gave their informed consent for inclusion before they participated in the study. The study was conducted in accordance with the Declaration of Helsinki. Control families (*n* = 10), comprised 37 individuals with a mean age 45.3 ± 18.4 (range 18.2 to 82.6 years) and were recruited with approval from the University of Leicester’s Research Ethics Committee (Ref: njr-61d3). Lynch syndrome families (*n* = 24) comprising 91 individuals with a mean age 47.0 ± 15.8 (range 19.2 to 73.1 years) were recruited in the Birmingham Women and Children’s Hospital (BWH) and the Leicester Royal Infirmary (LRI) under the ethical approval for the ‘Molecular Pathology of Human Genetic Disease (HumGenDis)’ study (REC reference CA/5175; and UHL10942/ IRAS 50895). Saliva samples were collected from two generations in each family with the older generation being designated as ‘parents’ and the younger generation as ‘children’. The one exception was the BWHF105 family, with one saliva sample also collect form a third generation. In this family, the individual BWHF105 G4P9 is the child in one comparison and the parent in another.

### 2.1. Sample Processing and Telomere Length Measurements

DNA was extracted from saliva samples using a DNA saliva kit (Oragene, Genotek, Ottawa, ON, Canada). The samples were coded and telomere length analyses conducted blind. STELA was used to amplify XpYp and 12q telomeres as described previously [20]. The analysis of individual chromosomes ends using STELA facilitates the detection of subtle effects on telomeres, for example the frequency of very short dysfunctional telomeres that might trigger cell senescence or subtle changes to length. The XpYp and 12q telomeres were selected because there is substantial evidence that the primers used in STELA are specific to these chromosomes ends. Primers designed to some other telomere-adjacent regions amplify from an unknown number of chromosome ends, which may also vary between individuals. Telomere length measurement using STELA from an unknown number of chromosome ends could complicate the comparative analysis in an unpredictable manner. Furthermore, we previously demonstrated that XpYp and 12q telomeres become unstable in MMR deficient cell lines and shorten at a faster rate than MMR-proficient cells [13]. In addition, XpYp STELA products from a lymphoblastoid cell line (KK) were included in all gels as a control for length analysis. Total abundance of telomeric repeats (T) was assessed by quantitative PCR (qPCR) using amplification of a single copy gene (S, RPLP0 (aka 36B4)) as a reference to report the T/S ratio for each sample [21]. The inter-run coefficient of variation for the qPCR measurements was 3.54%.

### 2.2. Statistical Analyses

Linear regression and other statistical analyses were conducted using Graph-Pad Prism, version 7.0. Age-adjusted telomere lengths (ΔTel) were calculated as described previously [17] and child/parent comparisons of the ΔTel age-adjusted values calculated. Distributions of ΔTel child-ΔTel parent values were compared using the non-parametric Mann–Whitney and Kruskal Wallis tests for two or more groups, respectively.

## 3. Results

High molecular weight DNA was extracted from the saliva samples collected from the control and LS families described in Table 1 and Figure S1. 

### 3.1. Cross-sectional Analysis of Total Telomeric DNA and Individual Telomere Lengths in LS and Controls

**Figure 1:**
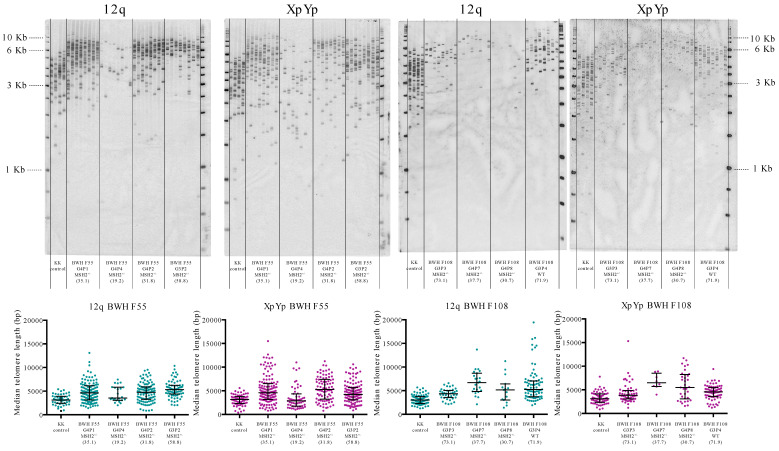
Examples of XpYp and 12q single telomere length analysis (STELA) in two LS families. Top panel, STELA southern blots for 12q and XpYp telomeres in four individuals from two families (BWH F55 and BWH108). XpYp STELA products from the KK cell line were included as a control. Bottom panel, scatter plots for quantification of median telomere length. Unique codes for each individual identify generation (G) and position in the pedigree (P). Numbers in brackets indicate the age (in years) at sample collection.

Median telomere lengths were measured in saliva DNA from control and LS families using STELA at XpYp and 12q (Figure 1) and total telomeric DNA abundance was assessed by T/S-qPCR. 

**Figure 2:**
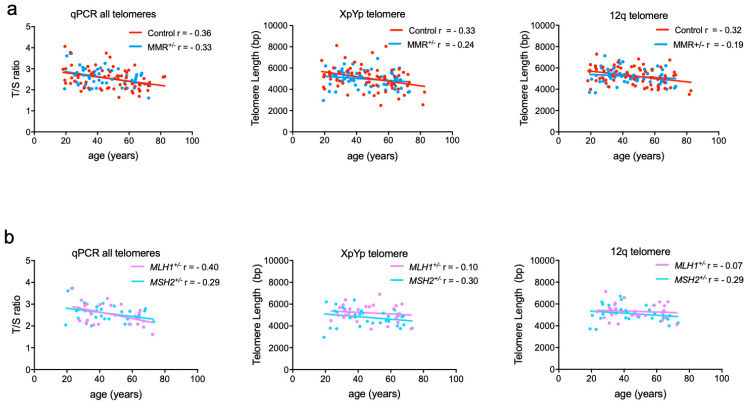
Graphs of telomere measurements versus age for control and Lynch syndrome cohorts. All graphs show telomere length measurements (Y axis) plotted against age (X axis) for each individual. (**a**) Shows the linear regression analysis for LS (MMR^+/−^) in blue (*n* = 60) and healthy controls in red (*n* = 68) for all (T/S-qPCR) telomeres, XpYp and 12q telomeres. (**b**) Shows comparison of the linear regression lines for *MHS2*^+/−^ in light blue (*n* = 33) and *MLH1*^+/−^ in mauve (*n* = 27) for all telomeres, XpYp and 12q telomeres. Spearman correlation values (r) are shown for all graphs.

In the control cohort, all three telomere measurements exhibited the expected decrease with age, and linear regression analyses showed slopes that were significantly different from zero (Figure 2a, Table S1). These cross-sectional analyses are consistent with the XpYp and 12q telomeres shortening at an average 21.8 ± 7.6 and 16.7 ± 5.6 bp per year, respectively, in the control cohort. The T/S-qPCR data from LS carriers (MMR^+/−^) also showed a significant decline with age. In contrast, the graphs of median XpYp and 12q telomere lengths versus age in the LS cohort showed lower r^2^ and r values and the slopes were not significantly different from zero (Figure 2a, Table S1). This suggests that the length dynamics at individual telomeres are different in MMR^+/−^ carriers, a feature that is masked in the qPCR data. Nevertheless, the linear regression slopes, which represent the cross-sectional age-related shortening, were not significantly different between the control and MMR^+/−^ cohorts (Table S1). To determine whether *MLH1* and *MSH2* mutations are associated with different effects on telomere length, regression analyses were conducted with the MMR^+/−^ cohort split by genotype. This also revealed very weak negative correlations between age and XpYp and 12q telomere lengths that were not significantly different from zero (Figure 2b; Table S1). The *MSH2^+/−^* mutation carriers tended to have shorter XpYp telomeres than the *MHL1^+/−^* carriers (Figure 2b), with a lower Y-intercept (*p* = 0.0427, Table S1), suggesting that *MSH2^+/−^* individuals start adulthood with some shorter telomeres. 

### 3.2. Comparison of Age-adjusted of Telomere Lengths within Families 

**Figure 3:**
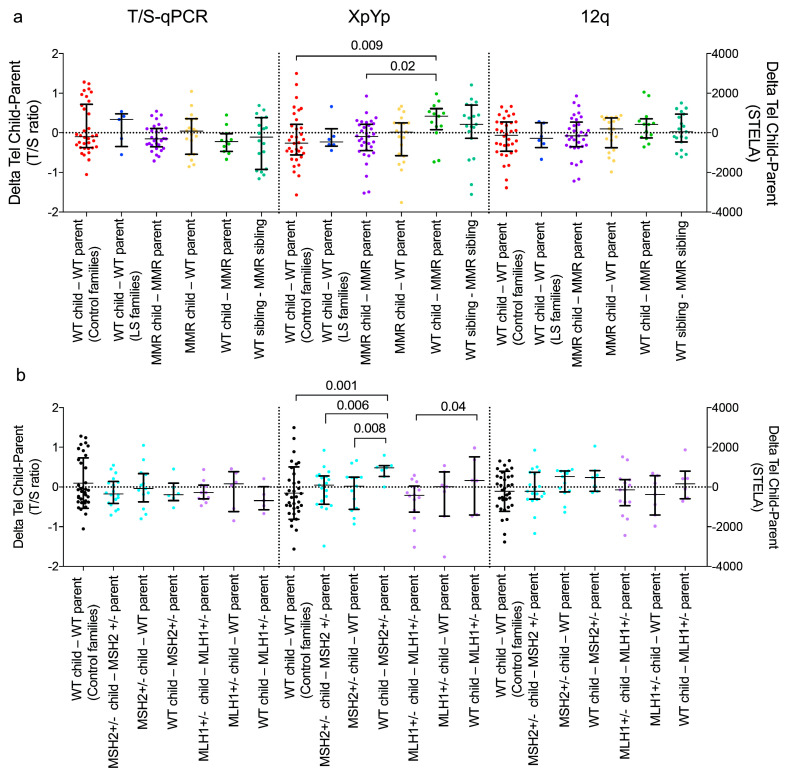
Comparison of age-adjusted telomere measurements within families. (**a**) Scatter plots of the difference between age-adjusted telomere measurements (ΔTel) for children and parents in the control and LS families and between WT and MMR+/– siblings in the LS families. The analysis is shown for the T/S-qPCR, XpYp and 12q data with median and interquartile ranges. (**b**) Scatter plots are shown for LS families with *MSH2* or *MLH1* mutations. The scatter plot distributions were compared using a non-parametric Kruskal Wallis test.

To investigate inter-generational differences in telomere length in LS and control families, telomere measurements were compared after adjusting for age (ΔTel) [17]. The difference in the aged-adjusted telomere measurements between a child and a parent (ΔTel_child_ - ΔTel_parent_, Table S2), showed that the MMR^+/−^ children did not have consistently shorter telomeres than either WT or MMR^+/−^ parent. In contrast, the WT children in LS families had significantly longer age-adjusted XpYp telomeres than their MMR^+/−^ parent (Figure 3a). A similar non-significant trend was seen at the 12q telomere but not in the T/S-qPCR data. Subdivision of the MMR^+/−^ families according to gene mutation (*MLH1*^+/−^ or *MSH2*^+/−^) reduced the number of child-parent comparisons that could be made, but it confirmed that WT and not *MSH2*^+/−^ or *MLH1*^+/−^ children tend to have longer XpYp telomeres than their *MSH2*^+/−^ or *MLH1*^+/−^ parent (Figure 3b).

## 4. Discussion

The aim of this study was to determine whether haplo-insufficiency for DNA MMR impacts on telomere length in heterozygous carriers of MMR gene mutations. The various methods for telomere length measurement each have advantages and disadvantages [22]. Therefore, we used complementary approaches including the T/S-qPCR method to assess total abundance of (TTAGGG)_n_ DNA and STELA to measure median lengths at the XpYp and 12q telomeres in saliva DNA samples from control and LS families [20,21].

The cross-sectional analyses of XpYp and 12q telomere lengths in the healthy controls showed significant shortening with age, in line with other studies [23,24]. In contrast, the same analysis in MMR^+/−^ carriers did not show a strong negative correlation between telomere length and age. This may suggest that there is a wider distribution of telomere lengths at any age among MMR^+/−^ carriers, which could be a consequence of MMR^+/−^ driven telomere instability. In addition, the cross-sectional and Y-intercept analyses showed that adult *MSH2^+/−^* carriers tended to have shorter XpYp telomeres, than controls and *MLH1*^+/−^ carriers. MSH2 is involved, as a heterodimer with MSH6 or MSH3, in the detection and binding to mismatches or insertion/deletion loops in DNA but also in the role that the MMR machinery plays in the cell cycle response to DNA damage. The data presented raise the possibility that MSH2 deficiency has a greater effect on telomere dynamics than MLH1 deficiency but the consequences may vary between telomeres. The question then arises as to why some telomeres might be more adversely affected by MSH2 deficiency than others. One possibility is variation in the composition and density of telomere variant repeats (TVRs) in the proximal regions of human telomeres, as this is known to vary considerably between chromosome ends, alleles and populations [8,12,25,26]. Differences in the extent of TVR composition between the XpYp and 12q telomeres is likely to influence the dynamics of telomere repeat turnover [8,12,26], as indicated in a comparative analysis of TVR abundance in LS and sporadic colorectal cancers [27]. 

The subtle differences we identified between individual telomeres indicate that telomere length dynamics differ between controls and MMR mutation carriers, particularly if *MSH2* is mutated but these differences were masked in the bulk analysis of all telomeres (using T/S-qPCR, Figure 2 and Table S1). STELA has the advantage that it detects short, potentially dysfunctional telomeres efficiently, but it is limited to the analysis of only a few telomeres, whereas T/S-qPCR measures mean (TTAGGG)_n_ abundance from all telomeres, but may under-represent very short or very long telomeres [28]. In this study, the intra-individual comparison of the XpYp and 12q telomere lengths measured by STELA, showed a stronger positive correlation than between individual telomere length measurements and the T/S-qPCR value (Figure S2), which probably reflects the different advantages and disadvantages of the methods.

For MMR haplo-insufficiency to contribute to genetic anticipation in LS families we proposed that MMR^+/−^ children would show shorter age-adjusted telomere length than their MMR^+/−^ parents. In fact, comparison of parent-child age-adjusted telomere lengths showed that MMR^+/−^ children did not have significantly shorter or longer telomeres than either parent. Consequently, we did not find evidence of an inter-generational effect on telomere length that might be attributed to shorter telomere length in gametes. In contrast, WT children had significantly longer age-adjusted XpYp telomeres than their MMR^+/−^ parents, with a stronger effect in *MSH2* families, and a similar non-significant trend at 12q. Moreover, *MSH2*^+/−^ carriers tend to start adulthood with shorter telomeres than WT individuals. Altogether the data presented here show there is a complex relationship between telomere instability and turnover of somatic cells during the lifetime of MMR^+/−^ carriers, in particular *MSH2*^+/−^ individuals.

## Figures and Tables

**Table 1:** Description of Lynch syndrome (LS) and control families.

	LS Families (N = 24)					Control Families (N = 10)		
	MMR Mutation-Carriers (N = 60)		Non-Carriers (N = 31)			Controls (N = 37)		
	Cancer	Cancer-free	Cancer	Cancer-free	Total 91	Cancer	Cancer-free	Total 37
n	32	28	2	29		3	34	
Parent: N (%)	24 (75)	^b^ 3 (11)	2 (100)	^c^ 15 (52)	44 (48)	3 (100)	15 (44)	18 (49)
Children: N (%)	8 (25)	25 (89)	0 (0)	14 (48)	47 (52)	0 (0)	19 (56)	19 (51)
MSH2^+/−^: N (%)	15 (47)	18 (64)	-	-	33 (36)	-	-	
MLH1^+/−^: N (%)	17 (53)	10 (36)	-	-	27 (30)	-	-	
^a^ MMR^+/+^: N (%)	-	-	2 (100)	29 (100)	31 (34)	3 (100)	34 (100)	37 (100)
Sex: N (%)	F: 14 (44)	F: 17 (61)	F: 2 (100)	F: 16 (55)	F: 49 (54)	F: 2 (67)	F: 21 (62)	23 (62)
	M: 18 (56)	M: 11 (39)	M: 0 (0)	M: 13 (45)	M: 42 (46)	M: 1 (33)	M: 13 (38)	14 (38)
Mean age at sample ±SD parents	60 ± 8	66 ± 2	65 ± 11	63 ± 7	62 ± 8	56 ± 3	62 ± 10	61 ± 10
Mean age at sample ±SD children	35 ± 8	33 ± 7	-	35 ± 8	34 ± 7	-	31 ± 11	31 ± 11
Mean age 1st cancer ±SD parents	45 ± 11	-	59 ± 9	-	-	44 ± 8	-	-
Mean age 1st cancer ±SD children	24 ± 7	-	-	-	-	-	-	-

Abbreviations: n: number of families or individuals; *N* (%): number of subjects and percentage within a group; ^a^ MMR^+/+^: homozygous for a wild type MMR gene; F: female; M: male. SD: standard deviation; ^b^ One *MSH2*^+/−^ individual in a three generation LS family was treated as child and as parent. ^c^ 15 MMR^+/+^ parents in the LS families were included as controls in age versus telomere length graphs (total controls = 52).

## Supplementary Materials

The following are available online at https://www.mdpi.com/2075-1729/10/11/265/s1, Figure S1: Pedigrees of families analyzed; Figure S2: Comparison of telomere length measurement methods; Table S1: Statistics associated with the cross-sectional analyses of telomere lengths in Figure 2; Table S2: Age-adjusted telomere measurements and comparisons between family members.
