# Supplementary material for: Telomere Instability in Lynch Syndrome Families Leads to Some Shorter Telomeres in MSH2+/- Carriers
Source: Life (Basel). 2020 Oct 31;10(11):265. doi: 10.3390/life10110265 (PMC7692680; doi:10.3390/life10110265)
Supplement: Supplementary file 1 [file life-10-00265-s001.zip › life-956855-supplementary/suplementary-for proof+NJR.docx]

Supplementary Material

Telomere Instability in Lynch Syndrome Families Leads to Some Shorter Telomeres in *MSH2+/−* Carriers


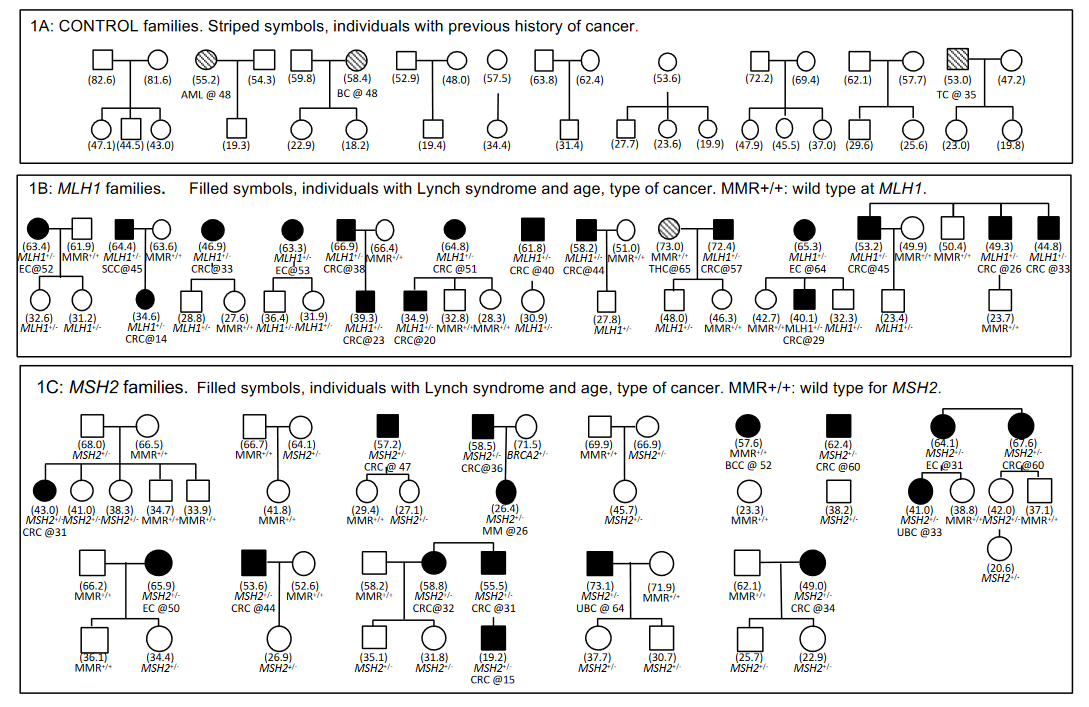


**Figure 1.** Families analysed. Age at sampling is shown in brackets. Age of first cancer is shown after @. Squares are males, circles are females.AML, acute myeloid leukaemia. BC, breast cancer. TC, testicular cancer. THC, thyroid carcinoma. EC: endometrial cancer. CRC: colorectal cancer. SCC: squamous cell carcinoma. UBC: urothelial carcinoma. MM: malignant melanoma. BCC: basal cell carcinoma.


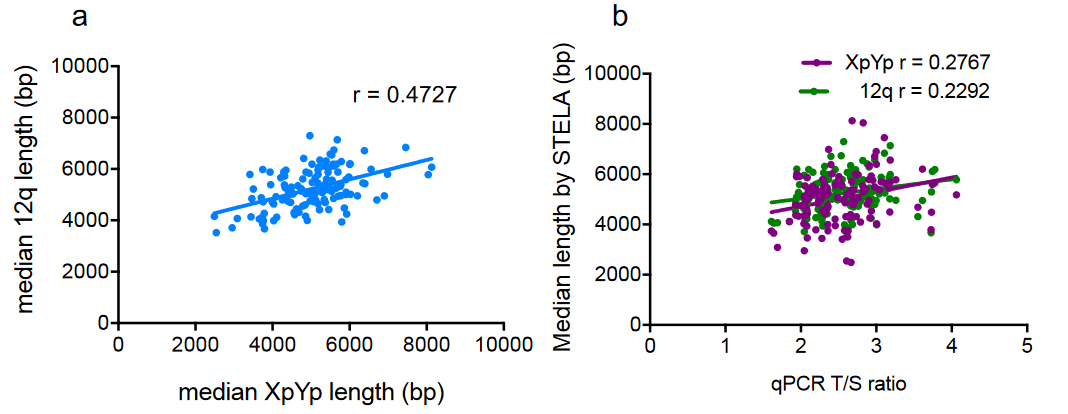


**Figure S2.** Comparison of telomere length measurement methods. (**a**) Correlation between median XpYpand 12q telomere lengths measured by STELA (**b**) Correlation between qPCR measurement of (TTAGGG)n abundance and median XpYpor 12q telomere length, measured by STELA.

**Table S1.** Statistics associated with the cross−sectional analyses of telomere lengths in figure 2.

| **Figure 2A** | | | | | | | |
| --- | --- | --- | --- | --- | --- | --- | --- |
|  |  | **qPCR Controls** | **qPCR MMR+/−** | **Median XpYp Controls** | **Median XpYp MMR+/−** | **Median 12q Controls** | **Median 12q MMR+/−** |
| Best-fit  values ± SE | Slope | −0.0100 ± 0.0034 | −0.0116 ± 0.0035 | −21.7500 ± 7.5990 | −9.7150 ± 6.6960 | −16.7200 ± 5.6070 | −7.2050 ± 6.0770 |
|  | Y−intercept | 3.0100 ± 0.1710 | 3.0930 ± 0.1681 | 6069 ± 378.7000 | 5420 ± 322.6000 | 6030 ± 279.5000 | 5533 ± 292.8000 |
| 95%  Confidence Intervals | Slope | −0.0168 to −0.0031 | −0.0187 to −0.0045 | −36.9200 to −6.5710 | −23.1200 to 3.6880 | −27.9200 to −5.5210 | −19.3700 to 4.9600 |
|  | Y−intercept | 2.6680 to 3.3510 | 2.7560 to 3.4300 | 5313 to 6825 | 4774 to 6066 | 5472 to 6589 | 4946 to 6119 |
| Goodness of  Fit | R square | 0.1174 | 0.1633 | 0.1119 | 0.0350 | 0.1203 | 0.0237 |
| Is slope significantly non-zero? | DFn. DFd | 1.6400 | 1. 5500 | 1. 6500 | 1. 58 | 1. 6500 | 1. 5800 |
|  | P value | 0.0049 | 0.0018 | 0.0057 | 0.1522 | 0.0040 | 0.2406 |
|  | Deviation from zero? | Significant | Significant | Significant | Not Significant | Significant | Not Significant |
|  | Equation | Y = −0.0100*X + 3.0100 | Y = −0.0116*X + 3.0930 | Y = −21.7500*X + 6069 | Y = −9.7150*X + 5420 | Y = −16.7200*X + 6030 | Y = −7.2050*X + 5533 |
| Correlation with age | Spearman r | −0.3591 | −0.3299 | −0.3300 | −0.2370 | −0.3151 | −0.1909 |
|  | P (two−tailed) | 0.0031 | 0.0122 | 0.0064 | 0.0682 | 0.0094 | 0.1441 |
|  | P value summary | ** | * | ** | Not Significant | ** | Not Significant |
| Are elevations or intercepts equal? | F | 0.0095 | | 0.3099 | | 0.1906 | |
|  | DFd | 120 | | 124 | | 124 | |
|  | P value | 0.9227 | | 0.5787 | | 0.6632 | |
|  | Pooled intercept | 3.0450 | | 5788 | | 5815 | |
| Figure 2B | | | | | | | |
|  |  | qPCR MLH1+/− | qPCR MSH2+/− | median XpYp MLH1+/− | median XpYp MSH2+/− | median 12q MLH1+/− | median 12q MSH2+/− |
| Best-fit  values ± SE | Slope | −0.0148 ± 0.0061 | −0.0094 ± 0.0045 | −7.6360 ± 10.4400 | −11.8700 ± 8.4280 | −4.4850 ± 9.6240 | −9.4750 ± 7.8850 |
|  | Y−intercept | 3.2300 ± 0.2900 | 3.0010 ± 0.2056 | 5554 ± 505.8000 | 5329 ± 404.2000 | 5529 ± 466.4000 | 5535 ± 378.1000 |
| 95%  Confidence Intervals | Slope | −0.0274 to −0.0022 | −0.0183 to −0.0005 | −29.1300 to 13.8600 | −29.0600 to 5.3200 | −24.3100 to 15.3400 | −25.5600 to 6.6060 |
|  | Y−intercept | 2.6300 to 3.8300 | 2.5810 to 3.4210 | 4512 to 6595 | 4505 to 6154 | 4569 to 6490 | 4764 to 6306 |
| Goodness of  Fit | R square | 0.2045 | 0.1346 | 0.0210 | 0.0601 | 0.0086 | 0.0445 |
| Is slope significantly non-zero? | F | 5.9140 | 4.6650 | 0.5354 | 1.9830 | 0.2172 | 1.4440 |
|  | DFn. DFd | 1. 2300 | 1. 3000 | 1. 2500 | 1. 3100 | 1. 2500 | 1. 3100 |
|  | P value | 0.0232 | 0.0389 | 0.4712 | 0.1690 | 0.6452 | 0.2386 |
|  | Deviation from zero? | Significant | Significant | Not Significant | Not Significant | Not Significant | Not Significant |
|  | Equation | Y = −0.0148*X + 3.2300 | Y = −0.0094*X + 3.0010 | Y = −7.6360*X + 5554 | Y = −11.8700*X + 5329 | Y = −4.4850*X + 5529 | Y = −9.4750*X + 5535 |
| Correlation with age | Spearman r | −0.3985 | −0.2898 | −0.0971 | −0.2975 | −0.0696 | −0.2940 |
|  | P (two-tailed) | 0.0485 | 0.1076 | 0.6300 | 0.0927 | 0.7301 | 0.0968 |
|  | P value summary | * | Not Significant | Not Significant | Not Significant | Not Significant | Not Significant |
| Are elevations or intercepts equal? | F | 0.0154 | | 4.2970 | | 1.4080 | |
|  | DFd | 54 | | 57 | | 57 | |
|  | P value | 0.9016 | | 0.0427* | | 0.2402 | |
|  | Pooled intercept | 3.092 | |  | | 5543 | |
